# Exome Evaluation of Autism-Associated Genes in Amazon American Populations

**DOI:** 10.3390/genes13020368

**Published:** 2022-02-18

**Authors:** Giovana E. da Costa, Giordane L. Fernandes, Juliana C. G. Rodrigues, Diana F. da V. B. Leal, Lucas F. Pastana, Esdras E. B. Pereira, Paulo P. Assumpção, Rommel M. R. Burbano, Sidney E. B. dos Santos, João F. Guerreiro, Marianne R. Fernandes, Ney P. C. dos Santos

**Affiliations:** 1 Núcleo de Pesquisas em Oncologia, Universidade Federal do Pará, Belém 66075-110, Brazil; giovanaescribano@hotmail.com (G.E.d.C.); giordanelages@hotmail.com (G.L.F.); julianacgrodrigues@gmail.com (J.C.G.R.); dianafeio@hotmail.com (D.F.d.V.B.L.); lucas.pastana@ics.ufpa.br (L.F.P.); rommelburbano@gmail.com (R.M.R.B.); sidneysantos@ufpa.br (S.E.B.d.S.); npcsantos.ufpa@gmail.com (N.P.C.d.S.); 2 Laboratório de Genética Humana e Médica, Instituto de Ciências Biológicas, Universidade Federal do Pará, Belém 66075-110, Brazil; esdrasedgarbp@gmail.com (E.E.B.P.); assumpcaopp@gmail.com (P.P.A.); joao.guerreiro53@gmail.com (J.F.G.)

**Keywords:** autism, susceptibility, genetic, Amerindians

## Abstract

Autism spectrum disorder is a neurodevelopmental disorder, affecting one in 160 children worldwide. The causes of autism are still poorly understood, but research shows the relevance of genetic factors in its pathophysiology, including the *CHD8*, *SCN2A*, *FOXP1* and *SYNGAP1* genes. Information about the genetic influence on various diseases, including autism, in the Amerindian population from Amazon, is still scarce. We investigated 35 variants of the *CHD8*, *SCN2A*, *FOXP1*, and *SYNGAP1* gene in Amazonian Amerindians in comparison with publicly available population frequencies from the 1000 Genomes Project database. Our study identified 16 variants in the Amerindian population of the Amazon with frequencies significantly different from the other populations. Among them, the *SCN2A* (rs17183814, rs75109281, and rs150453735), *FOXP1* (rs56850311 and rs939845), and *SYNGAP1* (rs9394145 and rs115441992) variants presented higher frequency than all other populations analyzed. In addition, nine variants were found with lower frequency among the Amerindians: *CHD8* (rs35057134 and rs10467770), *SCN2A* (rs3769951, rs2304014, rs1838846, and rs7593568), *FOXP1* (rs112773801 and rs56850311), and *SYNGAP1* (rs453590). These data show the unique genetic profile of the indigenous population of the Brazilian Amazon. Knowledge of these variants can help to understand the pathophysiology and diagnosis of autism among Amerindians, Brazilians, and in admixed populations that have contributions from this ethnic group.

## 1. Introduction

Autism spectrum disorder (ASD) is one of the main neuropsychiatric conditions and is characterized by different behavioral manifestations, including deficits in social communication and interaction, repetitive patterns of behavior, interests, and performance in specific activities [1]. The World Health Organization estimates that one in every 160 children is identified with ASD, being approximately 70 million people with ASD worldwide. In Brazil, it is estimated that 2 million people have ASD (1%) [2,3].

According to the Brazilian Institute of Geography and Statistics (IBGE), the Amerindian population is estimated at 896,917 individuals, representing 0.47% of the Brazilian population [4]. Studies about ASD in Amerindian populations are rare. In other countries, such as Australia, Amerindian Australians with the autism spectrum are twice as likely to have a severe or profound form of ASD and may have worse long-term outcomes compared to non-Amerindian Australians with the same condition. It was reported that Amerindians had less support and access to services in health and medicines [5]. 

ASD was discovered by Kanner in 1943 in the U.S. and by Asperger in 1944 in Vienna [6,7]. Currently, the causes of autism are still not well understood, although environmental, non-genetic, and genetic factors contribute to the disease. Bai et al. evaluated the contribution of various genetic and non-genetic factors to ASD risk. Researchers estimated heritability with maternal effects and shared and nonshared environments on ASD risk, including more than 2 million individuals from 5 countries. This study results reported significant evidence that most of the risk for ASD came from genetic factors [8,9].

In the largest genetic sequencing study of autism spectrum disorder (ASD) to date, researchers identified 102 genes related to the risk of ASD. The study enrolled 35,584 participant samples, including nearly 11,986 with ASD. Allelic variations in the 102 genes were related to susceptibility to neurodevelopmental disorders, such as ASD, and were able to differentiate this condition from other general neurodevelopmental disorders [10]. However, the impact of genetic factors associated with ASD in the Amazonian Amerindian is still unknown.

This is the first genetic study based on single-nucleotide polymorphisms (SNPs) associated with ASD in Amerindians from the Brazilian Amazon. This study characterizes the molecular profile of four of 102 genes related to ASD from a study by Satterstrom et al. [10] by analyzing the exome of Amerindian individuals from the Brazilian Amazon. The objective was to describe SNPs that may explain the predisposition to the development of ASD in Amazonian Amerindian and compare them with the worldwide population.

## 2. Materials and Methods

### 2.1. Study and Reference Populations

The Indigenous group (IND) was composed of non-related 64 Amerindians which represent 12 Amazonian ethnic groups of Northern Brazil: (i) Asurini do Xingu (N = 5), (ii) Arara (N = 7), (iii) Araweté (N = 6), (iv) Asurini from Tocantins (N = 16), (v) Awa-Guajá (N = 8), (vi) Kayapó/Xikrin (N = 2), (vii) Zo’é (N = 5), (viii) Wajãpi (N = 10), (ix) Karipuna (N = 1), (x) Phurere (N = 1), (xi) Munduruku (N = 1), and (xii) Juruna (N = 2). 

All participants of the study and their ethnic group leaders signed a free-informed consent. The study was approved by the National Research Ethics Committee (CONEP) and the Research Ethics Committee of the Tropical Medicine Center of the Federal University of Pará, under CAAE number 20654313.6.0000.5172. The period for recruiting participants was from September 2017 to December 2018. 

The results were compared with genomic data from populations from other countries available in the phase 3 version of the 1000 Genomes Database [11]. These populations included 661 Africans (AFR), 347 Americans (AMR), 504 from East Asia (EAS), 503 from Europe (EUR), and 489 from South Asia (SAS).

### 2.2. Extraction of the DNA and Preparation of the Exome Library

DNA extraction was performed using the phenol-chloroform method described by Sambrook et al. [12]. The quantification and integrity of the genetic material were analyzed by a Nanodrop-8000 spectrophotometer (Thermo Fisher Scientific Inc., Wilmington, DE, USA) and 2% agarose gel electrophoresis, respectively. 

Exome libraries were prepared using the Nextera Rapid Capture Exome (Illumina^®^, San Diego, CA, USA) and SureSelect Human All Exon V6 (Agilent) kits. The sequencing reactions were performed on the NextSeq 500^®^ platform (Illumina^®^, San Diego, CA, USA) using the NextSeq 500 High-output v2 300 cycle kit (Illumina^®^, San Diego, CA, USA).

### 2.3. Bioinformatics Analysis

Bioinformatics analysis was performed as previously described by Rodrigues et al. [13].

### 2.4. Statistical Analysis

Allele frequencies of the IND populations were obtained by gene counting compared to the other study populations (AFR, EUR, AMR, EAS, and SAS). Fisher’s test was used to compare frequency differences between populations. A *p*-value < 0.05 was considered statistically significant. Interpopulation variability of polymorphisms was assessed using the Wright fixation index (FST). Data analyses were performed using RStudio version 3.5.1.

### 2.5. Selection of Genes and Variants

The selection of genes was based in the results pointed out in the study of Satterstrom et al. [10]. Four of these genes, *CHD8*, *SCN2A*, *FOXP1,* and *SYNGAP1*, were classified as risk genes with the lowest rates of “false discovery rate (FDR)” and “family-wise error Rate (FWER)”.

The SNP inclusion criteria were: (i) minimum of 10 reeds of coverage (fastx_tools v.0.13 http://hannonlab.cshl.edu/fastx_toolkit/, accessed on October 2021); (ii) variant impact: modifier, moderate or high (SNPeff classification (https://pcingola.github.io/SnpEff/, accessed on October 2021); and (iii) allelic and genomic frequency in worldwide populations (http://www.1000genomes.org, accessed on October 2021).

## 3. Results

A total of 59 genetics variants were identified in *CHD8*, *SCN2A*, *FOXP1*, and *SYNGAP1* (Supplementary Table S1). Thirty-five of 59 variants met the SNP inclusion criteria. Eight variants were identified in the *CHD8* gene, eleven in *SCN2A*, twelve in *FOXP1*, and four in *SYNGAP1* in the individuals analyzed. Table 1 shows characteristics of these variants, including their reference number, chromosome region, nucleotide exchange, impact predicted by the SNPeff software, and the allele frequency referring to the indigenous group (IND) and the five continental populations present in the 1000 Genomes Program (AFR, AMR, EAS, EUR, and SAS) [11]. Among the selected polymorphisms, thirty have predicted impact as a modifier and five as moderate. Twenty-eight are from the intronic region, five from the CDS region, and two from the 3′UTR region. The frequencies of 35 variants were compared with different population groups (Table 2).

Among the 35 variants, 16 variants showed frequencies among Amerindians significantly different from all other populations. Seven variants with greater frequency among Amerindians: *SCN2A* (rs17183814, rs75109281, and rs150453735), *FOXP1* (rs939845 and rs2037474), and *SYNGAP1* (rs115441992 and rs9394145). Nine variants with lower frequency among the Amerindians: *CHD8* (rs35057134 and rs10467770), *SCN2A* (rs3769951, rs2304014, rs1838846, and rs7593568), *FOXP1* (rs112773801 and rs56850311), and *SYNGAP1* (rs453590).

The EUR and SAS populations stand out as those with the most variants with significant differences for the Amerindian population (*p* < 0.05). For the EUR population, five were in the *CHD8* gene (rs35057134, rs10467770, rs111776414, rs1998332, and rs149307240), nine in the *SCN2A* gene (rs17183814, rs75109281, rs3769951, rs28472553, rs139906774, rs2304014, rs150453735, rs1838846, and rs7593568), seven in the *FOXP1* gene (rs112773801, rs58847217, rs72960080, rs13068094, rs56850311, rs939845, and rs2037474), and four in *SYNGAP1* (rs76557362, rs453590, rs115441992, and rs9394145).

For SAS, four were in the *CHD8* gene (rs35057134, rs10467770, rs1998332, and rs149307240), nine were in the *SCN2A* gene (rs17183814, rs75109281, rs3769951, rs28472553, rs139906774, rs2304014, rs150453735, rs1838846, and rs7593568), eight in *FOXP1* (rs112773801, rs58847217, rs72960080, rs13068094, rs56850311, rs939845, rs2037474, and rs15101125) and four in *SYNGAP1* (rs76557362, rs453590, rs115441992, and rs9394145).

In relation to the AFR population, five polymorphisms were found to be significantly divergent in the *CHD8* gene (rs35057134, rs10467770, rs57764234, rs111776414, and rs149307240), eight in the *SCN2A* gene (rs17183814, rs75109281, rs3769951, rs2304014, rs150453735, rs1867864, rs1838846, and rs7593568), four in *FOXP1* (rs112773801, rs56850311, rs939845, and rs2037474), and four in the *SYNGAP1* gene (rs76557362, rs453590, rs115441992, and rs9394145), adding up to a total of twenty-one significantly different variants of the IND population.

The AMR population presented twenty-two statistically different polymorphisms in relation to the IND population: three in the *CHD8* gene (rs35057134, rs10467770, and rs1998332), nine in the *SCN2A* gene (rs17183814, rs75109281, rs3769951, rs28472553, rs139906774, rs2304014, rs150453735, rs1838846, and rs7593568), six in the *FOXP1* gene (rs112773801, rs72960080, rs13068094, rs568503111, rs939845, and rs2037474), and four in *SYNGAP1* (rs76557362, rs453590, rs115441992, and rs9394145).

The EAS population showed six statistically different variants in the *CHD8* gene (rs35057134, rs10467770, rs57764234, rs111776414, rs1998332, and rs149307240), nine in the *SCN2A* gene (rs17183814, rs75109281 rs3769951, rs28472553, rs139906774, rs2304014, rs150453735, rs1838846, and rs7593568), five in the *FOXP1* gene (rs112773801, rs58847217, rs72960080, rs56850311, and rs939845), and four in the *SYNGAP1* gene (rs76557362, rs453590, rs115441992, and rs9394145), summing twenty-four polymorphisms.

The rs35057134 (*CHD8*) had a low frequency in the Amerindian group, with differences greater than 20% of that found in the world population, as well as the rs10467770 (*CHD8*), rs3769951 (*SCN2A*), rs2304014 (*SCN2A*), and rs112773801 (*FOXP1*). Otherwise, the rs17183814 (*SCN2A*) variant presented higher frequencies in the Amerindian population, in contrast to those found in the world populations, except for EAS. This frequency pattern is also observed in the rs9394145 (*FOXP1*), which shows higher frequencies in Amazonian Amerindians.

**Figure 1:**
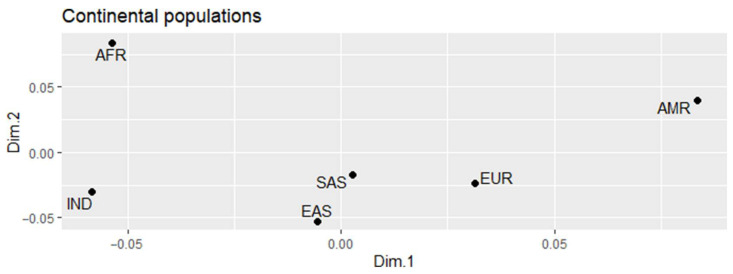
Multidimensional scale graph illustrating the ethnic populations grouping according to the genetic profile of the 35 variants in the *CHD8*, *SCN2A*, *FOXP1*, and *SYNGAP1* genes.

Multidimensional scale analysis (MDS), using FST values (Supplementary Table S2) for the 35 variants in the *CHD8*, *SCN2A*, *FOXP1*, and *SYNGAP1* genes revealed the existence of four major groups (Figure 1): The African population (AFR) is completely isolated, showing greater genetic diversity, as well as the American population (AMR); European (EUR), East Asian (EAS), and South Asian (SAS) populations clustered in the lower center; and the Indigenous group (IND) in the lower left corner. This analysis reported that the Amazonian population distances itself from other world populations concerning the variants analyzed for ASD susceptibility. The populations diverged significantly from African populations and showed greater proximity with populations from South and East Asia, compared to populations of European and, even, Latin American peoples.

## 4. Discussion

Previous evidence suggests ASD is modulated by genetic factors, such as SNPs. However, it is unclear which genes or SNPs contribute significantly to autism. A large genetic sequencing report showed 102 genes associated with the risk of autism [10]. In this study, we selected four genes from this previous study. We identified and characterized candidate SNPs in these selected genes associated with ASD, which have not been studied in Amazonian Amerindians. We also compared these data with worldwide populations. We hypothesize that SNPs in *CHD8*, *SCN2A*, *FOXP1*, and *SYNGAP1* genes could predispose an individual to ASD, especially those with a greater contribution of Amerindian ancestry.

The influence of ancestry difference in the autism spectrum disorder is limited. Population-based studies of the prevalence of autism spectrum disorder (ASD) in the United States have reported no differences among selected racial and ethnic groups, however without analyzing other ethnicities, such as native people [14].

There is still a lack of research investigating this issue, especially in Brazil. The Brazilian population has an admixture population characterized by a tetra-hybrid ancestry with European, African, American, and Asian composition [15]. Besides few genetic studies related to ASD in Brazil, there are no studies on this subject in Amerindians.

A previous study by Shochet et al. [16] had shown that Indigenous with ASD people living in remote areas had limited access to healthcare services. This is due to cultural and linguistic differences that are potential barriers to the diagnosis and treatment of this condition among the Amerindian population. Besides, some clinical features, such as avoiding eye contact and social communication, were not considered problematic in Amerindian cultures [17].

Current studies showed heritability of ASD was estimated to be approximately 50 to 80%, indicating that the variation in ASD occurrence in the population was mostly owing to inherited genetic influences [8,18]. Satterstrom and collaborators have identified 102 ASD risk genes in a large-scale genetic analysis to date. These genes, including *CHD8*, *SCN2A*, *FOXP1*, and *SYNGAP1*, regulate the development and function of the human brain [10].

The present study is the first to investigate the *CHD8*, *SCN2A*, *FOXP1*, and *SYNGAP1* genes in Amazonian Amerindians and highly admixed population in the Amazon region of Brazil with a major Amerindian component. The Amerindian ancestral contribution in the Brazilian population is 17%, except in the Amazon region which increases up to approximately 30%. In this area, the Amerindian ancestry population has the highest contribution in the country [18,19].

Besides the *SCN2A*, *FOXP1*, and *SYNGAP1* genes, *CHD8* variants are among the most replicated and common findings in ASD genetic studies. They are associated with the most common form of autism spectrum disorder, classic autism, along with macrocephaly, distinct dysmorphic facial features, and gastrointestinal disturbance [20,21]. Genetic variants in the *SCN2A* gene are also important in ASD; they can play a significant role in psychiatric disorders. They were associated with childhood seizures, epileptic encephalopathy, epileptic syndromes, as well as intellectual disability, and ASD without epilepsy [22,23].

The *FOXP1* gene has been implicated in neurodevelopmental disorders, such as ASD, and the *FOXP1* syndrome, in individuals with the presence of autistic spectrum disorder traits, intellectual disability, language impairment, and psychiatric characteristics [24,25]. In addition, the *SYNGAP1* gene is associated with several neurodevelopmental disorders, including non-syndromic intellectual disability and ASD, with symptoms that include encephalopathy, epilepsy, hypotonia, stereotyped behaviors, and aggression [23].

Of 59 variants found in the exome analysis made of the *CHD8*, *SCN2A*, *FOXP1*, and *SYNGAP1* genes, 35 variants could potentially be associated with the development of autistic spectrum disorder. Among the investigated variants, five of them had a moderate impact. They were all classified as CDS (coding sequence) and 30 variants had a modifier impact, 28 were intronic, and 2 were in the 3′UTR region.

In the present study, we compared the genetic variability of Amerindian populations from the Amazon region with five populations from the 1000 Genomes Project [11]. Our results about the comparison between ethnic groups revealed that the AFR group were isolated, with the greatest genetic difference from the AMR. This finding is consistent with the history of human populations in the world, in which the Amerindian and African groups represent the extremes of the evolutionary process [26].

Still, regarding the comparative results between ethnic groups, the lowest values of genetic differences with Amazonian Amerindians were observed in the population of East Asia (FST value = 0.00219). This result corroborates the hypothesis of the “Bering Strait”, an extension of land that joined Northeast Asia and North America [27].

The distancing of the IND and AMR groups was not expected (FST value = 0.07114); however, this analysis was only evaluated in the variants found for the investigated genes. The sample of the American population of the 1000 Genomes Project includes several countries in Latin America, such as Mexico, Peru, Colombia, and Puerto Rico, countries with Amerindian ancestral contributions that are heterogeneous among them, due to the different historical aspects of their formations and their degree of genetic mixing, which can explain the distance we found [11,28,29,30].

The identification of genetic variants associated with autism in the Amerindian population may favor the development of specific screening and diagnosis tools for this population, as well as for the Brazilian population and admixed populations, which have an important contribution of Amerindian ancestry in their constitution.

## 5. Conclusions

Our study was the first to investigate genes associated with autism in the Amazonian Amerindian, an understudied population that has a unique genetic profile. Our findings identify and characterized ASD-related SNPs, which could facilitate early disease testing and diagnosis, as well as early intervention in the Amerindian population and admixture populations with high contribution of Amerindian ancestry. This study may help better understand the biological mechanisms involved in the development of autism.

## Figures and Tables

**Table 1:** Description of variants in the *CHD8*, *SCN2A*, *FOXP1*, and *SYNGAP1* genes in the Indigenous group and continental populations (African, American, East Asia, European, and South Asia) described in the 1000 Genomes Project.

Gene	SNP ID	Region	Alleles	Impact Predicted by SNPeff	IND	AFR	AMR	EAS	EUR	SAS
*CHD8*	rs35057134	Intronic	GA > G	Modifier	0.0143	0.2250	0.2070	0.3480	0.2740	0.2550
*CHD8*	rs80311097	Intronic	C > A	Modifier	0.0000	0.0610	0.0010	0.0000	0.0010	0.0000
*CHD8*	rs10467770	CDS	C > T	Moderate	0.0781	0.2240	0.1900	0.3450	0.2450	0.2490
*CHD8*	rs111250264	CDS	G > A	Moderate	0.0086	0.0050	0.0000	0.0000	0.0000	0.0000
*CHD8*	rs57764234	Intronic	C > T	Modifier	0.0246	0.3160	0.0290	0.0000	0.0210	0.0050
*CHD8*	rs111776414	Intronic	G > GA	Modifier	0.0417	0.1610	0.0120	0.0010	0.0020	0.0110
*CHD8*	rs1998332	Intronic	G > A	Modifier	0.6172	0.5730	0.7780	0.8880	0.9060	0.9170
*CHD8*	rs149307240	CDS	C > T	Moderate	0.0259	0.0000	0.0160	0.0000	0.0010	0.0000
*SCN2A*	rs17183814	CDS	G > A	Moderate	0.2500	0.0210	0.0820	0.1380	0.0570	0.1420
*SCN2A*	rs75109281	Intronic	C > T	Modifier	0.0833	0.0120	0.0030	0.0000	0.0000	0.0000
*SCN2A*	rs3769951	Intronic	C > T	Modifier	0.0135	0.1640	0.2480	0.2610	0.2920	0.3310
*SCN2A*	rs28472553	Intronic	A > C	Modifier	0.0833	0.0290	0.0030	0.0000	0.0010	0.0000
*SCN2A*	rs139906774	Intronic	G > GA	Modifier	0.0000	0.0520	0.3000	0.3420	0.2420	0.1860
*SCN2A*	rs2304014	Intronic	T > A	Modifier	0.0270	0.2280	0.1330	0.1410	0.1760	0.1390
*SCN2A*	rs6432821	Intronic	T > C	Modifier	1.0000	0.9520	0.9970	1.0000	1.0000	0.9990
*SCN2A*	rs150453735	Intronic	C > T	Modifier	0.1852	0.0020	0.0530	0.0000	0.0000	0.0000
*SCN2A*	rs1867864	Intronic	C > T	Modifier	0.4453	0.6130	0.4600	0.3430	0.5640	0.4870
*SCN2A*	rs1838846	Intronic	A > G	Modifier	0.0000	0.7930	0.7940	0.7430	0.8300	0.6980
*SCN2A*	rs7593568	Intronic	A > G	Modifier	0.0000	0.7950	0.7950	0.7430	0.8300	0.6950
*FOXP1*	rs1435680522	3UTR	GT > G	Modifier	0.0000	0.0000	0.0000	0.0000	0.0000	0.0000
*FOXP1*	rs112773801	3UTR	G > GT	Modifier	0.0167	0.4240	0.1860	0.4510	0.1340	0.3290
*FOXP1*	rs58847217	Intronic	T > C	Modifier	0.0278	0.1010	0.0040	0.0000	0.0000	0.0000
*FOXP1*	rs76145927	CDS	T > C	Moderate	0.0000	0.0000	0.0060	0.0390	0.0030	0.0000
*FOXP1*	rs72960080	Intronic	T > C	Modifier	0.0833	0.1190	0.0040	0.0000	0.0000	0.0000
*FOXP1*	rs13068094	Intronic	C > T	Modifier	0.0833	0.1040	0.5400	0.0550	0.5750	0.2820
*FOXP1*	rs7638391	Intronic	G > T	Modifier	1.0000	0.9970	0.9650	1.0000	0.9230	0.9780
*FOXP1*	rs56850311	Intronic	A > T	Modifier	0.0000	0.3960	0.2520	0.1060	0.2890	0.2230
*FOXP1*	rs7639736	Intronic	C > A	Modifier	0.0000	0.0760	0.0560	0.0730	0.0130	0.0200
*FOXP1*	rs939845	Intronic	A > G	Modifier	0.3984	0.1630	0.2250	0.1110	0.0640	0.0440
*FOXP1*	rs2037474	Intronic	A > G	Modifier	0.5156	0.2720	0.3430	0.4360	0.1360	0.2450
*FOXP1*	rs151011253	Intronic	T > TA	Modifier	0.0139	0.0850	0.0560	0.0310	0.0540	0.0960
*SYNGAP1*	rs76557362	Intronic	C > T	Modifier	0.0833	0.2530	0.0130	0.0000	0.0000	0.0000
*SYNGAP1*	rs453590	Intronic	C > T	Modifier	0.0000	0.2700	0.4060	0.6410	0.3860	0.5430
*SYNGAP1*	rs115441992	Intronic	C > T	Modifier	0.0833	0.0140	0.0090	0.0000	0.0130	0.0020
*SYNGAP1*	rs9394145	Intronic	C > T	Modifier	0.5078	0.0130	0.3240	0.2500	0.3150	0.3220

IND. Indigenous; AFR. African; AMR. American, EAS. East Asia; EUR. European; SAS. South Asia; SAS. CDS. coding sequence.

**Table 2:** Comparison between the allelic frequency of the Indigenous population and continental populations (African, American, East Asia, European, and South Asia) described in the database of 1000 Genomes Project.

Gene	DbSNP	IND vs. AFR *	IND vs. AMR *	IND vs. EAS *	IND vs. EUR *	IND vs. SAS *
*CHD8*	rs35057134	**5.98 × 10^−6^**	**2.74 × 10^−5^**	**5.31 × 10^−10^**	**1.39 × 10^−7^**	**7.66 × 10^−7^**
*CHD8*	rs80311097	0.24887	0.28751	0.21283	0.21318	0.21826
*CHD8*	rs10467770	**0.00572**	**0.03028**	**2.97 × 10^−6^**	**0.00220**	**0.00138**
*CHD8*	rs111250264	0.30959	0.28751	0.21283	0.21318	0.21826
*CHD8*	rs57764234	**6.46 × 10^−8^**	1.00000	**0.03481**	0.64915	0.06785
*CHD8*	rs111776414	**0.01566**	0.07929	**0.00504**	**0.00507**	0.05415
*CHD8*	rs1998332	0.50781	**0.01151**	**4.40 × 10^−7^**	**3.06 × 10^−8^**	**6.23 × 10^−9^**
*CHD8*	rs149307240	**0.02173**	0.36125	**0.03481**	**0.03493**	**0.03665**
*SCN2A*	rs17183814	**1.19 × 10^−10^**	**0.00026**	**0.02585**	**5.70 × 10^−6^**	**0.04046**
*SCN2A*	rs75109281	**0.00339**	**0.00042**	**8.63 × 10^−5^**	**8.70 × 10^−5^**	**9.84 × 10^−5^**
*SCN2A*	rs3769951	**0.00037**	**1.60 × 10^−6^**	**4.39 × 10^−7^**	**3.69 × 10^−8^**	**2.16 × 10^−9^**
*SCN2A*	rs28472553	0.05210	**0.00042**	**8.63 × 10^−5^**	**8.70 × 10^−5^**	**9.84 × 10^−5^**
*SCN2A*	rs139906774	0.35386	**3.54 × 10^−8^**	**6.13 × 10^−10^**	**1.54 × 10^−6^**	**0.00011**
*SCN2A*	rs2304014	**3.85 × 10^−5^**	**0.01857**	**0.00941**	**0.00166**	**0.01459**
*SCN2A*	rs6432821	0.10268	1.00000	1.00000	1.00000	1.00000
*SCN2A*	rs150453735	**9.91 × 10^−13^**	**0.00066**	**1.86 × 10^−11^**	**1.90 × 10^−11^**	**2.57 × 10^−11^**
*SCN2A*	rs1867864	**0.01590**	1.00000	0.09628	0.10907	0.69027
*SCN2A*	rs1838846	**8.09 × 10^−38^**	**3.83 × 10^−35^**	**5.39 × 10^−32^**	**1.00 × 10^−40^**	**2.50 × 10^−28^**
*SCN2A*	rs7593568	**5.55 × 10^−38^**	**3.83 × 10^−35^**	**5.39 × 10^−32^**	**1.00 × 10^−40^**	**3.54 × 10^−28^**
*FOXP1*	rs1435680522	0.16887	0.28751	0.21283	0.21318	0.21826
*FOXP1*	rs112773801	**2.15 × 10^−13^**	**0.00013**	**3.53 × 10^−14^**	**0.00344**	**2.18 × 10^−9^**
*FOXP1*	rs58847217	0.07415	0.06453	**0.03481**	**0.03493**	**0.03665**
*FOXP1*	rs76145927	0.16887	0.39900	0.49517	0.30231	0.21827

IND. Indigenous; AFR. African; AMR. American, EAS. East Asia; EUR. European; SAS. South Asia; SAS. CDS. coding sequence. *. Fisher’s exact test.

## Data Availability

The dataset used in this study is publicly available. The name of the repository and accession number(s) can be found at https://doi.org/10.6084/m9.figshare.18822272, accessed on October 2021.

## Supplementary Materials

The following supporting information can be downloaded at: https://www.mdpi.com/article/10.3390/genes13020368/s1. Table S1: Description of all variants in the *CHD8*, *SCN2A*, *FOXP1*, and *SYNGAP1* genes found in the Amerindian individuals (IND) Table S2: Pairwise FST among Amerindians (IND) and the five continental populations from the 1000 Genomes Project.
