# Supplementary material for: Exome Evaluation of Autism-Associated Genes in Amazon American Populations
Source: Genes (Basel). 2022 Feb 18;13(2):368. doi: 10.3390/genes13020368 (PMC8871861; doi:10.3390/genes13020368)
Supplement: Supplementary file 1 [file genes-13-00368-s001.zip › genes-1543775-supplementary.pdf]

SUPPLEMENTARY TABLE S1 - DESCRIPTION OF ALL VARIANTS OF THE 5 GENES ANALYZED IN AMERINDIAN INDIVIDUALS (IND).

Gene	SNP ID	Region	Nucleotide change	Impact	IND	AFR	AMR	EAS	EUR	SAS
FOXP1	rs1435680522	3UTR	GT>G	MODIFIER	0	-	-	-	-	-
FOXP1	rs112773801	3UTR	G>GT	MODIFIER	0,01666667	0,42	0,19	0,45	0,13	0,33
FOXP1	rs9855825	intronic	A>T	MODIFIER	-	0,44	0,66	0,85	0,68	0,55
FOXP1	rs58847217	intronic	T>C	MODIFIER	0,02777778	0,1	0	0	0	0
FOXP1	rs76145927	CDS	T>C	MODERATE	0	0	0,01	0,04	0	0
FOXP1	rs760260048	CDS	G>A	LOW	0,00862069	0	0	0	0	0
FOXP1	rs72960080	intronic	T>C	MODIFIER	0,08333333	0,12	0	0	0	0
FOXP1	rs13068094	intronic	C>T	MODIFIER	0,08333333	0,1	0,54	0,06	0,58	0,28
FOXP1	rs17008224	intronic	G>A	MODIFIER	-	0	0,03	0,2	0,02	0,05
FOXP1	rs7638391	intronic	G>T	MODIFIER	1	1	0,97	1	0,92	0,98
FOXP1	rs56850311	intronic	A>T	MODIFIER	0	0,4	0,25	0,11	0,29	0,22
FOXP1	rs1295692908	CDS	A>T	MODERATE	0,08333333	0	0	0	0	0
FOXP1	rs7639736	intronic	C>A	MODIFIER	0	0,08	0,06	0,07	0,01	0,02
FOXP1	rs939845	intronic	A>G	MODIFIER	0,3984375	0,16	0,23	0,11	0,06	0,04
FOXP1	rs2037474	intronic	A>G	MODIFIER	0,515625	0,27	0,34	0,44	0,14	0,25
FOXP1	rs747208306	intronic	G>A	MODIFIER	0,0952381	0	0	0	0	0
FOXP1	rs151011253	intronic	T>TA	MODIFIER	0,01388889	-	-	-	-	-
SYNGAP1	rs76557362	intronic	C>T	MODIFIER	0,08333333	0,25	0,01	0	0	0
SYNGAP1	rs1245444837	intronic	C>CA	MODIFIER	-	-	-	-	-	-
SYNGAP1	rs453590	intronic	C>T	MODIFIER	0	0,27	0,41	0,64	0,39	0,54
SYNGAP1	rs115441992	intronic	C>T	MODIFIER	0,08333333	0,01	0,01	0	0,01	0
SYNGAP1	rs544817923	CDS	A>G	LOW	0,0703125	0	0	0	0	0
SYNGAP1	rs411136	CDS	G>A	LOW	0,375	0,27	0,4	0,63	0,38	0,54
SYNGAP1	.	CDS	C>G	MODERATE	0	-	-	-	-	-
SYNGAP1	rs9394145	intronic	C>T	MODIFIER	0,5078125	0,01	0,32	0,25	0,32	0,32
SYNGAP1	rs138475712	intronic	TTC>T	MODIFIER	0	-	-	-	-	-
CHD8	rs8022395	CDS	C>T	LOW	0,640625	0,95	0,81	0,89	0,93	0,92
CHD8	rs35057134	intronic	GA>G	MODIFIER	0,01428571	0,23	0,21	0,35	0,27	0,26
CHD8	rs201856289	CDS	A>T	LOW	0,08333333	0	0	0	0	0
CHD8	rs80311097	intronic	C>A	MODIFIER	0	0,06	0	0	0	0
CHD8	rs1021755285	intronic	A>G	MODIFIER	0	-	-	-	-	-
CHD8	rs931605748	CDS	T>C	LOW	0	0	0	0	0	0
CHD8	rs992446349	CDS	G>A	LOW	0	0	0	0	0	0
CHD8	rs10467770	CDS	C>T	MODERATE	0,078125	0,22	0,19	0,35	0,25	0,25
CHD8	rs111250264	CDS	G>A	MODERATE	0,00862069	0,01	0	0	0	0
CHD8	rs57764234	intronic	C>T	MODIFIER	0,02459016	0,32	0,03	0	0,02	0,01

CHD8	.	intronic	AAATGT>A	MODIFIER	0	-	-	-	-	-
CHD8	rs111776414	intronic	G>GA	MODIFIER	0,04166667	0,16	0,01	0	0	0,01
CHD8	rs1998332	intronic	G>A	MODIFIER	0,6171875	0,57	0,78	0,89	0,91	0,92
CHD8	rs113076568	intronic	C>CT	MODIFIER	0,11904762	0	0,76	0,87	0,87	0,01
CHD8	rs929799421	CDS	C>G	MODERATE	0,01724138	0	0	0	0	0
CHD8	rs149307240	CDS	C>T	MODERATE	0,02586207	0	0,02	0	0	0
SCN2A	rs17183814	CDS	G>A	MODERATE	0,25	0,02	0,08	0,14	0,06	0,14
SCN2A	rs1864885	intronic	A>G	MODIFIER	-	0,03	0,3	0,34	0,25	0,19
SCN2A	rs2060198	CDS	T>A	LOW	0	0,16	0,25	0,25	0,29	0,33
SCN2A	rs75109281	intronic	C>T	MODIFIER	0,08333333	0,01	0	0	0	0
SCN2A	rs3769951	intronic	C>T	MODIFIER	0,01351351	0,16	0,25	0,26	0,29	0,33
SCN2A	rs28472553	intronic	A>C	MODIFIER	0,08333333	0,03	0	0	0	0
SCN2A	rs3835933	intronic	TG>T	MODIFIER	0,22222222	-	-	-	-	-
SCN2A	rs145662546	CDS	C>T	LOW	0,08333333	0,01	0,01	0	0	0
SCN2A	rs139906774	intronic	G>GA	MODIFIER	0	0,05	0,3	0,34	0,24	0,19
SCN2A	rs2304014	intronic	T>A	MODIFIER	0,02702703	0,23	0,13	0,14	0,18	0,14
SCN2A	rs6432821	intronic	T>C	MODIFIER	1	0,95	1	1	1	1
SCN2A	rs150453735	intronic	C>T	MODIFIER	0,18518519	0	0,05	0	0	0
SCN2A	rs1867864	intronic	C>T	MODIFIER	0,4453125	0,61	0,46	0,34	0,56	0,49
SCN2A	rs1838846	intronic	A>G	MODIFIER	0	0,79	0,79	0,74	0,83	0,7
SCN2A	rs1361108483	intronic	A>G	MODIFIER	0	-	-	-	-	-
SCN2A	rs2121371	OTHER	T>C	LOW	0	0,79	0,79	0,74	0,83	0,7
SCN2A	rs7593568	intronic	A>G	MODIFIER	0	0,8	0,8	0,74	0,83	0,7

SUPPLEMENTARY TABLE S2 – PAIRWISE FTS BETWEEN THE AMERINDIANS (IND) AND THE FIVE CONTINENTAL POPULATIONS OF THE 1000 GENOMAS DATABASE.

	IND	AFR	AMR	EAS	EUR	SAS
IND	0.00000					
AFR	0.03729	0.00000				
AMR	0.07114	0.07615	0.00000			
EAS	0.00219	0.08752	0.05710	0.00000		
EUR	0.01505	0.09089	0.01324	0.07895	0.00000	
SAS	0.01693	0.07190	0.04242	0.02395	0.04951	0.00000
